# Multidetector Computed Tomography (MDCT) Angiography Evaluation of Total Anomalous Pulmonary Venous Connection

**DOI:** 10.7759/cureus.46852

**Published:** 2023-10-11

**Authors:** Priyadharshini Bala V, Deepak Barathi S, Ramkumar Govindarajalou, Selvaganesan M

**Affiliations:** 1 Radiodiagnosis, Jawaharlal Institute of Postgraduate Medical Education and Research, Puducherry, IND

**Keywords:** pulmonary veins, mdct angiography, cardiac ct, congenital heart disease, total anomalous pulmonary venous connection (tapvc)

## Abstract

Total anomalous pulmonary venous connection (TAPVC) is a rare congenital cardiovascular malformation in which all four pulmonary veins anomalously drain into the right atrium (RA) either directly or indirectly. There are four main types based on the site of connection. Any type of TAPVC may be associated with obstruction and presents early in the neonatal period with cyanosis, tachycardia, or respiratory distress. We present four cases of all types of TAPVC and its imaging findings in multidetector computed tomography (MDCT) angiography. Cardiac CT and magnetic resonance imaging (MRI) are very useful in delineating the anatomy and drainage pathway of anomalous pulmonary veins. MDCT angiography is noninvasive and easily available, and rapid image acquisition is possible with high spatial resolution. Since early diagnosis and surgical correction are necessary for the survival of these neonates, rapid image acquisition using MDCT angiography can be preferred over MRI.

## Introduction

Total anomalous pulmonary venous connection (TAPVC) is an uncommon congenital cardiovascular malformation in which all four pulmonary veins fail to drain into the left atrium and anomalously drain into the right atrium (RA) or to any of the systemic veins either directly or indirectly [1]. The incidence of this condition is approximately 7-9 per 100,000 live births [2,3], and it accounts for 1%-5% of all congenital heart diseases (CHD) [4]. There are four main types based on the site of connection. Right-to-left shunt in the form of a patent foramen ovale or an atrial septal defect (ASD) is almost always present so that an admixture of oxygenated and unoxygenated blood eventually reaches the left side of the heart, without which the infant would not survive [1].

In the evaluation of TAPVC, chest radiography, echocardiography, catheter angiography, multidetector computed tomography (MDCT) angiography, and magnetic resonance imaging (MRI) are useful. Due to recent advances in technology, MDCT angiography has become a reliable and noninvasive tool for diagnosing and planning treatment for pulmonary vein anomalies [5].

We report MDCT angiography findings of four cases of TAPVC: supracardiac type, cardiac type, mixed type (supracardiac and intracardiac), and a rare form of infracardiac type of TAPVC draining into the portal vein.

Cardiac CT procedure

All patients were imaged using a 128-slice MDCT (Somatom Definition Edge Scanner, Siemens Healthineers, Erlangen, Germany) in our institution. Patients were adequately sedated, if needed, and positioned in a caudocranial position with arms above the head, and electrocardiogram (ECG) leads were appropriately connected. The retrospectively gated non-contrast phase was done with as small field of view (FOV) as possible. Intravenous nonionic iodinated contrast medium was then administered (iohexol (Contrapaque) 300 mg) using a dual-head power injector at a flow rate of 1-1.5 mL/second for children and 5 mL/second for adults and a volume calculated as (delay + scan time) × flow rate (not more than 600 mg I/kg body weight). CARE Dose 4D (Siemens Medical Solutions, Forchheim, Germany) allowed for further minimization of radiation dose while maintaining adequate resolution for anatomical delineation. Images were analyzed in a cardiac CT post-processing workstation using Syngo.via version VB60A_HF01 (Siemens Healthcare, Erlangen, Germany), which generates multiplanar reconstruction (MPR) and volume rendering technique (VRT) images for additional aid.

## Case presentation

Case 1

**Figure 1:**
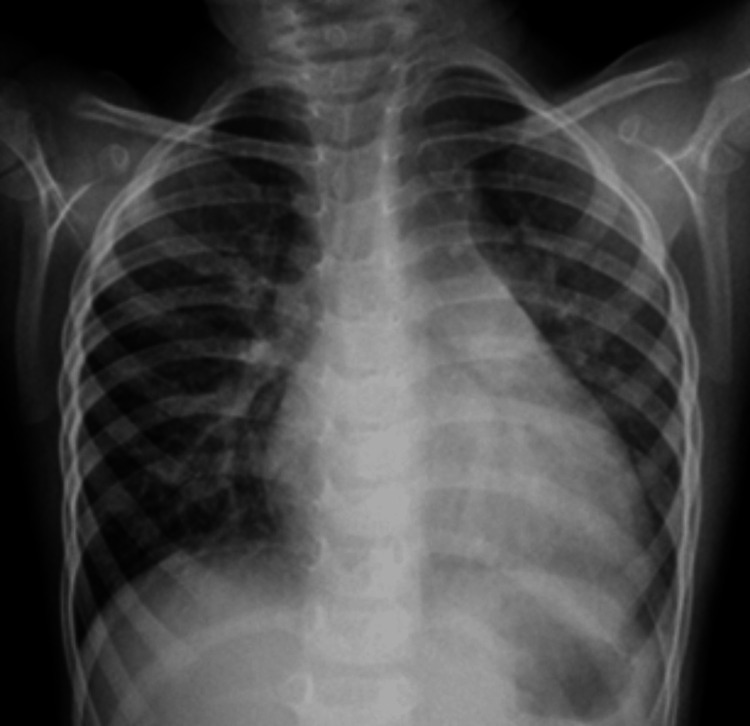
Plain chest radiography frontal view showing cardiomegaly with widening of the superior mediastinum

**Figure 2:**
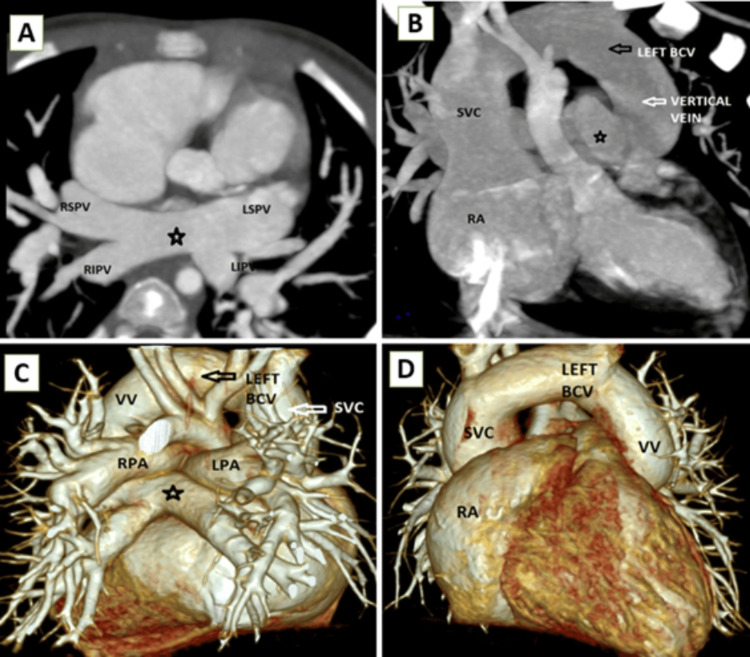
Supracardiac TAPVC (A) cardiac CT axial MIP sections showing confluence (black star) of all four pulmonary veins, (B) CT coronal post-contrast MIP section showing confluence of all four pulmonary veins (black star) joining to form a vertical vein draining into the SVC through the left brachiocephalic vein (LEFT BCV), and (C and D) 3D VR CT reconstructed images viewed from the posterior aspect (C) and anterior aspect (D) demonstrating anomalous drainage of all pulmonary veins into a dilated VV and SVC indirectly to the RA TAPVC: total anomalous pulmonary venous connection, MIP: maximum intensity projection, CT: computed tomography, SVC: superior vena cava, VR: volume rendered, VV: vertical vein, RA: right atrium, RSPV: right superior pulmonary vein, RIPV: right inferior pulmonary vein, LSPV: left superior pulmonary vein, LIPV: left inferior pulmonary vein, LEFT BCV: left brachiocephalic vein

**Figure 3:**
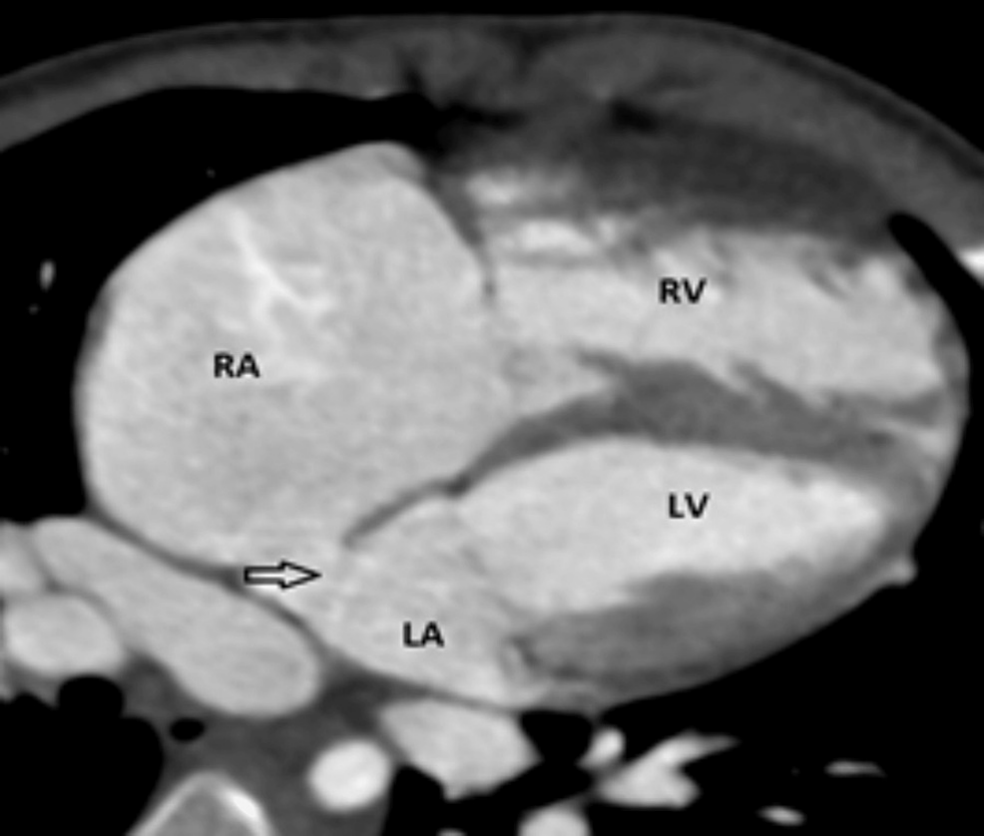
Cardiac CT axial section showing dilated RA and RV; there is also an atrial septal defect (black arrow) resulting in a right-to-left shunt CT: computed tomography, RA: right atrium, RV: right ventricle, LA: left atrium, LV: left ventricle

A two-year-and-six-month-old child with an unremarkable birth history had recurrent episodes of upper respiratory tract infection. On examination, she was found to have a systolic murmur. Chest radiograph findings were mild cardiomegaly with widened superior mediastinum (Figure 1). Echocardiography findings were a dilated right atrium and right ventricle, an ostium secundum atrial septal defect (OS-ASD), a right-to-left shunt, a common chamber seen posterior to the left atrium, and a vertical vein seen adjacent to the pulmonary artery (PA) that were suspicious of supracardiac TAPVC. Cardiac CT showed all four pulmonary veins joined and continued cranially as the vertical vein, which drained into the left brachiocephalic vein (BCV), representing a supracardiac type of total anomalous pulmonary venous connection (Figure 2A-2D). There is an associated ostium secundum ASD along with a dilated right atrium and right ventricle, as seen in Figure 3. Catheter angiography confirmed supracardiac TAPVC with drainage via the vertical vein into the left brachiocephalic vein and superior vena cava (SVC). There is no pulmonary arterial hypertension (PAH).

Case 2

**Figure 4:**
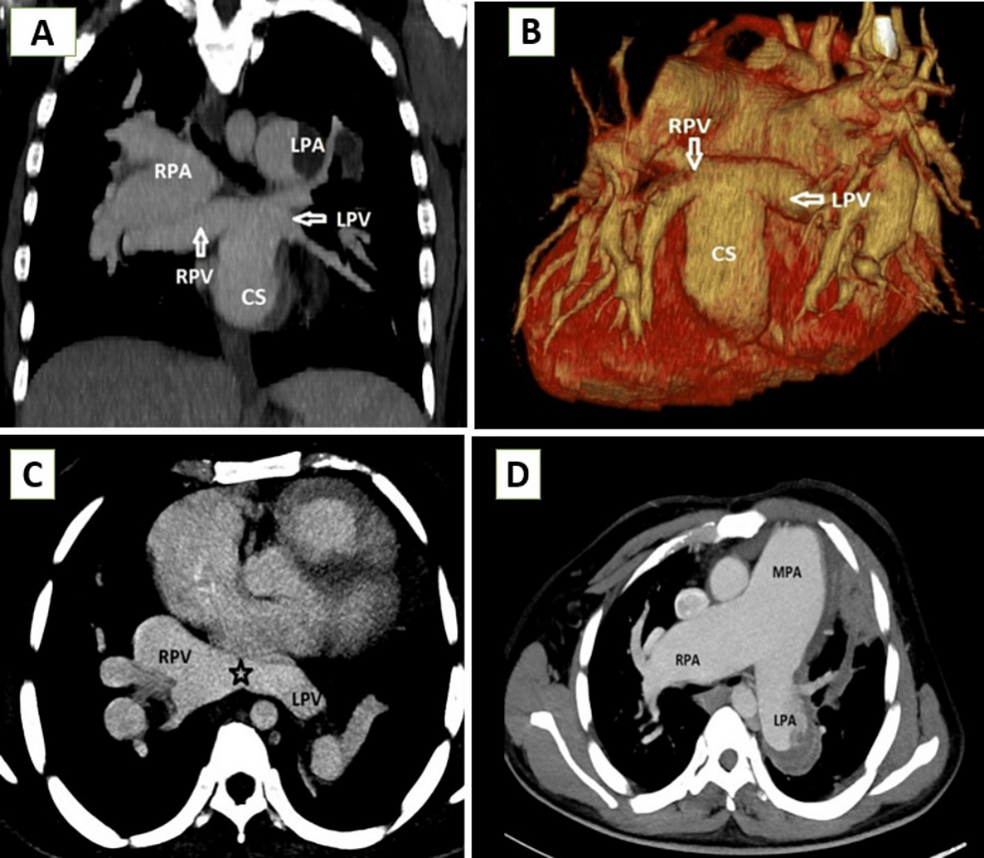
Intracardiac TAPVC (A and B) CT pulmonary angiography coronal MIP section (A) and 3D VR (B) images showing confluence of all four pulmonary veins draining into the dilated CS, (C) post-contrast axial MIP image showing confluence (black star) of all four pulmonary veins, and (D) post-contrast axial MIP image showing dilated MPA, RPA, and LPA and chronic thromboembolism involving the LPA and its upper lobar branches with infarct in the lingula TAPVC: total anomalous pulmonary venous connection, MIP: maximum intensity projection, VR: volume rendered, CS: coronary sinus, MPA: main pulmonary artery, RPA: right pulmonary artery, LPA: left pulmonary artery, RPV: right pulmonary vein, LPV: left pulmonary vein

A 30-year-old male patient, who was diagnosed with congenital heart disease 15 years ago, has been under medical management and has presented with complaints of shortness of breath for three days. On examination, the patient had central cyanosis, a generalized constant murmur, and coarse inspiratory crepitations in bilateral lung fields. A chest radiograph showed cardiomegaly with pulmonary plethora. Echocardiography findings were suspicious for intracardiac TAPVC and ASD. CT pulmonary angiography revealed all four dilated pulmonary veins forming a confluence (Figure 4C) and draining into a dilated coronary sinus (CS) entering the right atrium (Figure 4A, 4B). An associated large ASD with a dilated right atrium was also present. The main pulmonary trunk, along with the left and right pulmonary arteries, appears dilated with distal pruning due to pulmonary arterial hypertension (Figure 4D). Chronic left pulmonary thromboembolism with pulmonary infarcts involving the lingula was also present (Figure 4D). The patient was started on anticoagulants and advised to have corrective surgery later.

Case 3

**Figure 5:**
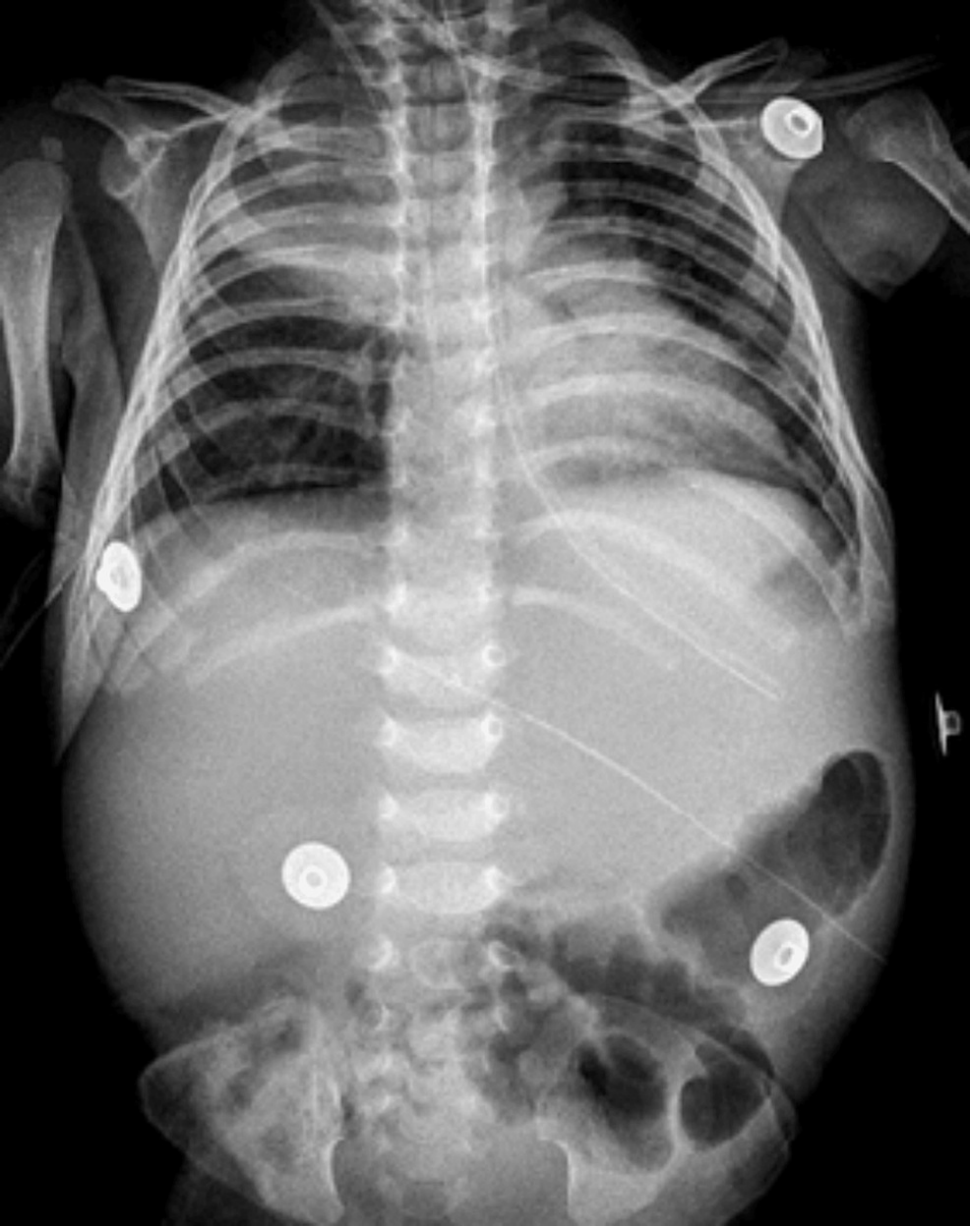
Plain chest and abdominal radiograph frontal view showing non-homogeneous opacities in the right upper lung zone with prominent pulmonary vasculature and enlarged hepatic silhouette with displacement of bowel loops

**Figure 6:**
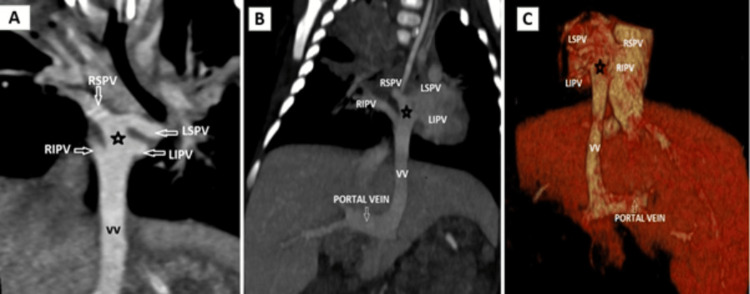
Infracardiac TAPVC (A) cardiac CT coronal MIP image showing the confluence of all pulmonary veins (black star) joining to form a common descending VV and (B and C) post-contrast coronal MIP and 3D VR images showing the vertical vein coursing through the diaphragmatic hiatus and draining into the dilated main portal vein TAPVC: total anomalous pulmonary venous connection, MIP: maximum intensity projection, CT: computed tomography, VV: vertical vein, VR: volume rendered, RSPV: right superior pulmonary vein, RIPV: right inferior pulmonary vein, LSPV: left superior pulmonary vein, LIPV: left inferior pulmonary vein

A two-month-old child presented to the emergency department with tachypnea, hypotension, and mild cyanosis. The child had a generalized constant heart murmur, slight pulmonary rales, and mild hepatomegaly on examination. A chest and abdominal radiograph showed non-homogeneous opacities in the right upper lung zone, with prominent pulmonary vasculature, an apparently normal cardiac silhouette, and an enlarged liver shadow, as seen in Figure 5. An echocardiogram revealed ASD with a right-to-left shunt, dilated right heart cavities, and a posterior chamber behind the left atrium receiving all four pulmonary veins with a dilated inferior vena cava (IVC). Cardiac CT showed bilateral upper and lower pulmonary veins joining to form a common descending vertical vein (Figure 6A), which coursed inferiorly through the esophageal hiatus and drained into the portal vein (Figure 6B, 6C). Also, an associated sinus venosus ASD and dilated right atrium and right ventricle were present. Patchy areas of consolidation involving bilateral lung fields were also noted. The patient underwent emergency surgery and unfortunately succumbed after the procedure due to multiple complications.

Case 4

**Figure 7:**
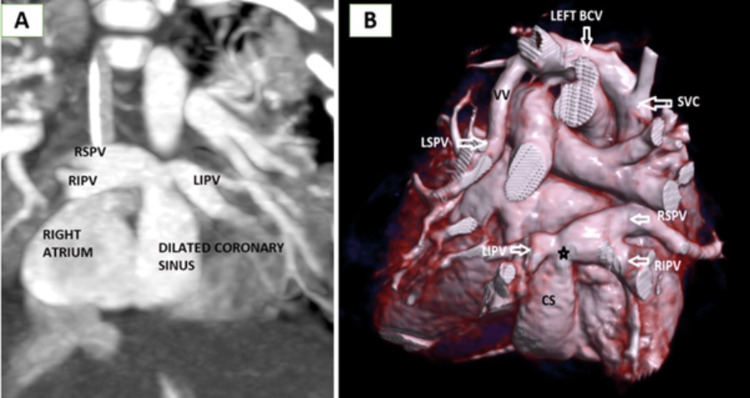
Mixed TAPVC (A and B) cardiac CT coronal MIP (A) and 3D VR (B) image showing RSPV and RIPV forming confluence with LIPV and joining the dilated CS and (B) coronal 3D reconstruction showing intracardiac drainage of the RSPV and bilateral inferior pulmonary veins draining into the dilated CS and LSPV coursing cranially to drain into the LEFT BCV via a VV, resulting in both supracardiac and intracardiac anomalous pulmonary venous connections TAPVC: total anomalous pulmonary venous connection, MIP: maximum intensity projection, CT: computed tomography, VR: volume rendered, RSPV: right superior pulmonary vein, RIPV: right inferior pulmonary vein, LIPV: left inferior pulmonary vein, LSPV: left superior pulmonary vein, CS: coronary sinus, LEFT BCV: left brachiocephalic vein, VV: vertical vein

A one-month-old child presented with respiratory distress, poor feeding, and lethargy. The child had bradycardia and hypokalemia and was mechanically ventilated in view of desaturation. Echocardiography showed two pulmonary veins draining into a dilated coronary sinus with OS-ASD and grossly dilated right-sided cavities. Cardiac CT revealed the right upper and bilateral lower pulmonary veins forming a confluence and draining into the coronary sinus (Figure 7A). The left upper pulmonary vein continues cranially as the vertical vein drains into the brachiocephalic vein (Figure 7B), representing a mixed (intracardiac and supracardiac) type of total anomalous pulmonary venous connection. Also, there was ostium secundum ASD, dilated pulmonary arteries, and bilateral lung consolidation. The child underwent emergency corrective surgery, and in the immediate postoperative period, he developed a PAH crisis and died of multiple complications.

## Discussion

Total anomalous pulmonary venous return (TAPVR), also known as total anomalous pulmonary venous connection, represents 1%-5% of cardiovascular congenital anomalies [4]. This type of congenital cardiovascular malformation is more frequently seen in patients with heterotaxy syndromes, especially asplenia or polysplenia [6,7]. It can also be associated with other complex cardiac and great vessel anomalies such as transposition of the great arteries, pulmonary stenosis, double outlet right ventricle, and coarctation of the aorta.

Embryology of pulmonary veins

The pulmonary buds develop from the foregut and are initially surrounded by the splanchnic plexus. This plexus drains into the systemic venous system, specifically the cardinal and umbilical vitelline veins. During gestation at 27-29 days, the primitive left atrium gives rise to the primitive common pulmonary vein. This vein connects to the lungs and establishes connections with the pulmonary portion of the splanchnic plexus, forming the pulmonary plexus. The pulmonary venous plexus becomes disconnected from the systemic circulation by detaching from the splanchnic plexus. Four individual pulmonary veins, two on each side, develop and drain to the common pulmonary vein. The four pulmonary vessels eventually drain into the left atrium through an orifice located on the posterior wall [8]. The persistence of connections between the primitive pulmonary veins and cardinal systemic veins results in TAPVC [9].

TAPVC is classified into four categories based on the sites where the abnormal connection occurs [10], namely, (1) supracardiac, (2) cardiac, (3) infracardiac, and (4) mixed type.

The supracardiac type is the most common type, accounting for 45%-55% of TAPVC cases. In this type, the confluent vessel usually empties into the innominate vein or the right or left superior vena cava [7,11] as in Figure 2A-2D. The confluent vein while coursing cranially may be extrinsically compressed by adjacent intrathoracic structures and presents with features of obstruction.

The cardiac type is the second common type presenting in 20%-30% of cases. It is diagnosed when the pulmonary veins converge to form a common vessel and then horizontally connect to the right atrium (RA) via the coronary sinus (CS) (Figure 4A, 4B) or at the posterior wall of the RA.

The infracardiac type is the third most common type. The pulmonary veins join to form a vertical vessel that travels caudally through the esophageal hiatus and drains into the IVC, portal vein, hepatic veins, or ductus venosus. This type accounts for 13%-25% of cases. In case 3, the vertical vein is seen draining into the portal vein as seen in Figure 6B, 6C. It is one of the most common types to present with obstruction while passing through the diaphragmatic hiatus or at the site where it joins with the systemic venous circulation.

The mixed type is the least common type with less than 10% of the cases, in which the right and left pulmonary tributaries drain at two or more different levels [8,12]. In our case 4, the right upper and bilateral lower pulmonary veins are seen draining into the coronary sinus. The left upper lobe vein drains into the left brachiocephalic vein. Hence, it is a case of mixed (cardiac and supracardiac) TAPVC (Figure 7A, 7B).

Clinical manifestation

Patients' presenting complaints vary depending on the severity of the obstruction and the resistance of the pulmonary vessels. In case of severe obstruction, neonates present within the first 12 hours of life with manifestations such as dyspnea, tachypnea, tachycardia, hypoxemia, and metabolic acidosis. It is necessary to perform surgical correction within the first few days of life for neonates to survive [8]. Patients without venous obstruction are typically asymptomatic at birth but may develop tachypnea, mild cyanosis, and feeding difficulties in the first few weeks. Gradual onset of recurrent respiratory tract infections and failure to thrive is observed in most patients, with only a few surviving into late childhood or adolescence without treatment. In patients with interatrial communication such as atrial septal defect (ASD) or patent foramen ovale, the size of the communication is important as it determines the amount of blood that enters the left heart and supplies the systemic circulation of infants [13].

Investigations

The details of the anatomy and drainage of the pulmonary veins are essential for proper preoperative planning [14]. Chest radiographs and echocardiography are initial investigations of choice [4,6,15]. Echocardiography can delineate the anatomy and defects in most cases; however, its role may be limited because of the small field of view and poor acoustic window [16,17]. MDCT angiography and magnetic resonance angiography (MRA) are highly valuable for mixed and infracardiac types. They provide precise anatomical information for presurgical planning and postsurgical follow-up [4,7,16,18]. Computed tomography allows for the performance of three-dimensional (3D) reconstructions for advanced spatial and anatomical orientation [7,16]. The advantages of MDCT comprise noninvasive, easily available, rapid image acquisition that can accurately show the drainage site, stenosis of the vertical vein, and course of the anomalous vessel with 100% sensitivity and specificity [7,19]. The use of a low-dose protocol based on neonatal body weight parameters can reduce ionizing radiation, which is the only disadvantage of MDCT [7]. The advantages of cardiac magnetic resonance imaging over MDCT include a lack of ionizing radiation and multiplanar capability. However, the need for sedation, prolonged scan times, susceptibility to motion-related artifacts, and the need for trained technicians may be disadvantages, favoring the use of MDCT [4,14].

Catheter angiography has been considered the gold standard for diagnosis, but it is an invasive and expensive method that requires hospitalization [14]. It does not provide information on the vessel's three-dimensional anatomical course or its relation to adjacent structures. It also fails to provide information on the vessel's 3D anatomical course and its relation to nearby structures. It can be used as an adjunctive tool along with cardiac CT before surgical repair.

Management

Accurate diagnosis is crucial for effective treatment, which typically involves surgical intervention. Improvements in diagnostic imaging have resulted in the accurate depiction of this complex entity, and advanced surgical techniques have decreased postoperative mortality rates [4]. The main surgical objective is to establish a normal connection between the pulmonary veins and the left atrium. Patients with obstructed pulmonary veins and other complex cardiac defects, including heterotaxy syndrome, may experience a worse outcome [8].

Table 1 summarizes the types of total anomalous pulmonary venous connection (TAPVC) [8,20].

**Table 1:** Summary of the types of TAPVC The mixed type is a combination of any two types of TAPVC. TAPVC: total anomalous pulmonary venous connection, ASD: atrial septal defect, PDA: patent ductus arteriosus, PFO: patent foramen ovale, CS: coronary sinus, RA: right atrium, RV: right ventricle, CT: computed tomography, MRI: magnetic resonance imaging

Types	Supracardiac	Cardiac	Infracardiac
Pathway of pulmonary venous return	All four pulmonary veins drain into the left brachiocephalic vein and superior vena cava	Common pulmonary vein drains into the RA or CS	The descending vertical vein passes through the diaphragm and drains into the inferior vena cava, portal vein, ductus venosus, or hepatic vein
Incidence [8]	45%-55%	20%-30%	13%-25%
Association	ASD, PDA, PFO, and heterotaxy syndrome		
Imaging findings	X-ray: snowman sign or figure of "8" configuration, pulmonary plethora	X-ray: cardiomegaly with RA/RV dilatation, pulmonary plethora	X-ray: normal or mild cardiomegaly, pulmonary edema
	CT: anatomy and drainage details of the common vertical vein to the left brachiocephalic vein	CT: anomalous pulmonary venous drainage into the dilated CS	CT: dilated descending vein passing through the diaphragm and draining into systemic veins
	MRI: hemodynamic assessment	MRI: functional assessment	MRI: postoperative evaluation
Management [20]	Ligation of the vertical vein and anastomosis of the left atrium and common pulmonary venous pathway	TAPVC to CS: unroofing of the CS with closure of the ostium of the CS and ASD	Ligation of the common channel and anastomosis of the common pulmonary vein above the diaphragm with the left atrium

## Conclusions

We have briefly described the embryology and imaging presentation of all four types of TAPVC with case illustration. Both MRI and cardiac CT angiography can provide precise anatomy and drainage details of pulmonary veins, which is essential for preoperative planning, especially in mixed and infracardiac types of TAPVC. Cardiac CT is noninvasive and easily available and allows rapid image acquisition for evaluating critically ill neonates and infants.
